# Prognostic and Predictive Determinants of Colorectal Cancer: A Comprehensive Review

**DOI:** 10.3390/cancers16233928

**Published:** 2024-11-23

**Authors:** Horia T. Cotan, Radu A. Emilescu, Cristian I. Iaciu, Cristina M. Orlov-Slavu, Mihaela C. Olaru, Ana M. Popa, Mariana Jinga, Cornelia Nitipir, Oliver Daniel Schreiner, Romeo Cristian Ciobanu

**Affiliations:** 1 General Medicine Faculty, Carol Davila University of Medicine and Pharmacy, 8 Sanitary Heroes Boulevard, 050474 Bucharest, Romania; horia-teodor.cotan@rez.umfcd.ro (H.T.C.); cristian-ion.iaciu@rez.umfcd.ro (C.I.I.); orlov.cristina@gmail.com (C.M.O.-S.); miha0611@yahoo.com (M.C.O.); amy.popa@yahoo.com (A.M.P.); mariana_jinga@yahoo.com (M.J.);; 2 Regional Institute of Oncology Iasi, 2-4 General Henri Mathias Berthelot Street, 700483 Iasi, Romania; oliver090598@yahoo.com; 3 Department 3—Medical Sciences, Grigore T. Popa University of Medicine and Pharmacy, 16 University Street, 700115 Iasi, Romania; 4 Department of Electrical Measurements and Materials, Gheorghe Asachi Technical University, 700050 Iasi, Romania

**Keywords:** prognostic marker, predictive markers, biomarkers, molecular targeted treatment

## Abstract

Colorectal cancer (CRC) remains a significant global health burden, necessitating a thorough understanding of prognostic and predictive factors to enhance patient outcomes. This systematic review aims to comprehensively evaluate prognostic and predictive determinants in CRC, encompassing both traditional and emerging biomarkers. A systematic search of major electronic databases was conducted to identify relevant studies published from 1995 up to 2024. Eligible articles were critically appraised, and data extraction was performed according to predefined criteria. The prognostic determinants examined included clinicopathological features such as tumor stage, grade, and lymph node involvement, as well as molecular biomarkers including RAS, BRAF, and MSI status. Predictive determinants encompassed biomarkers influencing response to targeted therapies and immunotherapy, such as HER2 and Immunoscore. The review also explores novel prognostic and predictive markers, including tumor microenvironment characteristics and liquid biopsy-based biomarkers. Synthesizing evidence from diverse studies, this review provides insights into the prognostic and predictive landscape of CRC, highlighting the potential clinical implications of identified determinants. Understanding the multifaceted nature of prognostic and predictive factors in CRC is imperative for the advancement of personalized treatment strategies and improvement of patient outcomes.

## Simple Summary

This systematic review explores prognostic and predictive factors in colorectal cancer, aiming to identify key determinants that influence patient outcomes and treatment responses. By synthesizing evidence from relevant studies, the review assesses the impact of various factors such as tumor characteristics, biomarker expression, and treatment modalities on disease prognosis and therapeutic efficacy. Through a comprehensive analysis, this review provides valuable insights into the factors that can help clinicians tailor treatment strategies and improve patient outcomes in colorectal cancer management.

## 1. Introduction

Colorectal cancer (CRC) is one of the most frequently occurring types of cancer, with an annual incidence of almost 2 million cases and a likewise high annual mortality of 935.000 cases [1]. Moreover, the total number of cases could increase to nearly 2.5 million by 2035 [2]. For CRC in the early stages (stages I to III), the primary treatment method is surgical removal. Currently, the most effective way to evaluate prognosis after curative surgery is through pathological examination of the removed tissue. While factors determining the pathological stage are the most significant indicators of the outcome, other clinical, molecular, and histological characteristics can also affect prognosis, regardless of stage. In patients with stage IV CRC, the prognosis is more closely associated with the location and extent of disease spread to distant areas as well as novel molecular and histological indicators.

While historically fluorouracil (5-FU) based therapies the standard in CRC treatment, the therapeutic landscape is currently rapidly evolving, concomitant with the approval of several targeted therapies [3]. This new and continuously evolving therapeutic landscape is a strong incentive for identifying cancer and host-specific biomarkers that will aid in the optimal therapeutic agent selection for each patient. The range of biomarkers employed in testing is steadily expanding. The National Institute of Health describes a biomarker as a biological molecule present in blood, other bodily fluids, or tissues, which indicates a normal or abnormal process, or the presence of a specific condition or disease. A biomarker is commonly defined as encompassing elements like DNA, RNA, microRNA (miRNA), epigenetic alterations, or antibodies. The term “tumor marker”, often used interchangeably with “biomarker”, encompasses a broad range of substances within the body, typically proteins or glycolipids, which serve as biological indicators. These markers hold significance as they are intricately associated with the intricate processes of both normal cellular development and the pathogenesis of cancer, spanning various stages of cell development. In the context of normal cell development, tumor markers play essential roles as biological signals guiding cellular growth, differentiation, and function. They are involved in maintaining cellular homeostasis and orchestrating physiological processes necessary for tissue integrity and function. These markers can indicate the presence of specific cellular activities or states, reflecting the dynamic nature of cellular processes within the body. However, in the context of carcinogenesis, tumor markers take on added significance as they become dysregulated or aberrantly expressed during the development and progression of cancer. Changes in the expression levels or patterns of tumor markers can signify alterations in cellular behavior, such as uncontrolled proliferation, evasion of apoptosis, angiogenesis, invasion, and metastasis, which are hallmarks of cancer development. For example, tumor-associated antigens (TAAs) constitute the most significant group of clinically relevant markers [4]. Consequently, the concentration of TAAs often directly correlates with the quantity (or mass) of specific cancer cells.

Although not considered biomarkers or tumor markers, pathologic features are significant prognostic determinants of CRC both in clinical practice as well as in research. Genetic assessments of tumor biopsies have become integral to the personalization of CRC treatment. By identifying specific genetic mutations and molecular characteristics, clinicians can tailor therapies to target these alterations, thereby improving treatment efficacy and patient outcomes. Both European and NCCN (National Comprehensive Cancer Network) guidelines have integrated genetic insights into their recommendations for CRC management, reflecting the contemporary shift towards precision medicine. The European Society for Medical Oncology (ESMO) guidelines recommend routine testing for MMR status, KRAS, NRAS, and BRAF mutations in all patients with metastatic CRC. These genetic assessments inform the selection of appropriate targeted therapies and immunotherapies, aligning treatment choices with the molecular characteristics of the tumor [3]. The NCCN guidelines similarly emphasize the importance of molecular testing in CRC. They recommend that all patients with metastatic CRC undergo testing for MMR/MSI status, as well as KRAS, NRAS, and BRAF mutations. These guidelines support the use of targeted therapies and immunotherapies based on the genetic profile of the tumor [5].

In this review, we assess the practical value of both established and emerging biomarkers currently in use or under investigation. These biomarkers play a crucial role in informing treatment decisions both in patients with metastatic colorectal cancer (mCRC) as well as those who develop resistance to treatment.

## 2. Materials and Methods

**Figure 1:**
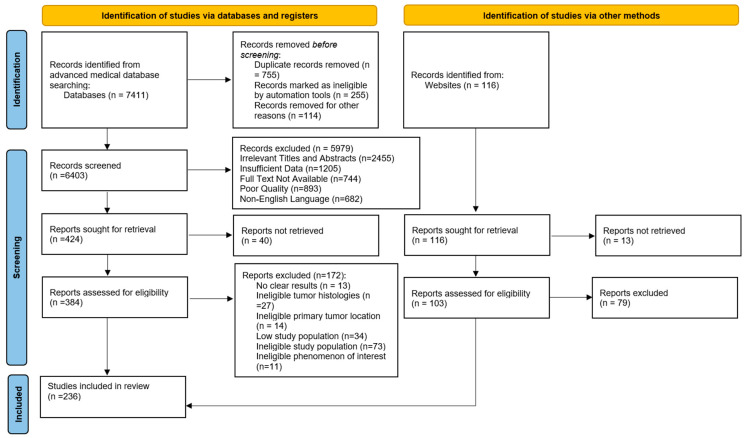
Consort flow diagram.

For this systematic review on prognostic and predictive determinants of colorectal cancer, the inclusion and exclusion criteria were carefully defined to ensure the relevance and quality of the included studies. We included studies that focused on identifying and evaluating prognostic and predictive factors in patients diagnosed with colorectal cancer. Eligible studies were peer-reviewed articles, clinical trials, cohort studies, case–control studies, and observational studies published in English between 1995 and 2024. Participants were adults (≥18 years) diagnosed with colorectal cancer, irrespective of the cancer stage at diagnosis. Interventions of interest included any treatment modality (surgery, chemotherapy, radiotherapy, targeted therapy, immunotherapy) with a focus on their prognostic or predictive impact. Comparators included studies with control groups or different treatment groups allowing for comparative analysis of prognostic and predictive outcomes. Key outcomes assessed were overall survival, disease-free survival, progression-free survival, and recurrence rates. Studies were excluded if they were non-human research, reviews, meta-analyses, editorials, letters, case reports, or if they lacked sufficient data on prognostic or predictive factors. Additionally, studies focusing solely on genetic or molecular markers without clear clinical correlation were excluded to maintain a clinical focus. We conducted a systematic literature search to locate relevant studies and publications. This search utilized the PubMed database (www.ncbi.nlm.nih.gov/pubmed/ accessed on 16 June 2024) and the Cochrane Library (https://www.cochranelibrary.com/advanced-search accessed on 16 June 2024) with specific search terms and criteria: either “colorectal cancer”, “metastatic colorectal cancer” or “mCRC” combined with “biomarkers”, “prognostic markers” or “molecular biomarkers” in the title or abstract. We focused exclusively on English-language publications, particularly those concerning clinical trials, meta-analyses, observational studies, comparative studies, clinical studies, systematic reviews, multicenter studies, or case reports, published between 1 January 1995, and 1 January 2024. The search yielded 7.411 results. We meticulously reviewed the titles and abstracts of these articles, and for those reporting on emerging biomarker data in CRC, we obtained and closely examined the full-text versions. Additionally, a manual search on ClinicalTrials.gov was performed to identify ongoing clinical trials that are exploring emerging biomarkers in CRC or are focused on molecular-guided clinical trials. The process of study selection is shown in Figure 1. This review was performed in accordance with the PRISMA (Preferred Reporting Items for Systematic Reviews and Meta-Analyses) guidelines and has not been registered.

## 3. Pathologic Features

### 3.1. Bowel Obstruction and/or Perforation

While all colorectal cancers (CRCs) initially develop from adenomas or flat dysplasia, their gross appearance undergoes diverse morphological changes during the course of invasion and expansion. In the proximal (right) colon, tumors typically exhibit the appearance of a polypoid or fungating exophytic mass. This presentation may lead to clinical manifestations such as occult bleeding, contributing to an unexplained iron deficiency anemia. In contrast, tumors affecting the distal (left) colon more frequently present as annular or encircling lesions, resulting in an observable “apple-core” or “napkin-ring” appearance on imaging. These encircling lesions are more commonly associated with manifestations of bowel dysfunction, such as constipation, diarrhea, or bowel obstruction. When clinical bowel obstruction occurs or perforation of the bowel wall is present, it generally leads to a poorer prognosis [6,7]. That being said, several studies did not report an adverse prognostic impact for clinical obstruction at the time of diagnosis in CRC [8,9,10].

Gross perforation is generally recognized as an adverse prognostic factor in CRC according to most studies [11,12,13,14,15,16,17,18], although a few reports [8,10,19] do not support this conclusion. While numerous studies have found that obstruction and/or perforation independently predict poorer survival outcomes in multivariate analysis, some research suggests that CRCs requiring emergency surgery due to complications like obstruction or perforation often display a more aggressive histopathological profile. This manifests as more advanced disease stage and unfavorable histological characteristics, compared to cases where surgery can be electively planned.

Obstruction and perforation are categorized as clinicopathologic factors that identify “high-risk” stage II colon cancer, according to guidelines from major oncology organizations. These include the National Comprehensive Cancer Network (NCCN), the European Society for Medical Oncology (ESMO), and the American Society of Clinical Oncology (ASCO). This categorization underscores the significance of these conditions in influencing prognosis and potential treatment strategies for stage II colon cancer.

### 3.2. Tumor Location

Despite differences in their gross appearance, right- and left-sided colon cancers are microscopically similar and appear to have a comparable prognosis when presenting with locoregional disease [20]. However, in cases of metastatic disease, some evidence suggests that patients with a right-sided primary tumor may have a worse prognosis [21,22]. Most but not all [23] studies agree that primary tumor location is a significant prognostic factor in CRC [24,25,26,27,28]. In a comprehensive meta-analysis comprising 66 studies and encompassing 1,427,846 patients across all disease stages, the presence of a left-sided primary tumor (located at or beyond the splenic flexure) was correlated with a significantly lower risk of mortality (hazard ratio [HR] 0.82, 95% CI 0.79–0.84). Importantly, this association remained consistent irrespective of disease stage, though the effect magnitude was more pronounced for metastatic disease. Furthermore, factors such as race, utilization of adjuvant chemotherapy, year of study, and the quality of the included studies did not alter the observed relationship between left-sided tumor location and reduced mortality risk [26].

Tumor location may serve as a surrogate for underlying molecular biology. For instance, in a particular study, mutations in BRAF or KRAS, linked to a less favorable prognosis, were found to be more prevalent in proximal (right-sided) cancers. In contrast, distal (left-sided) tumors tended to be nonmutated. This suggests that the molecular profile associated with specific genetic mutations may vary based on the anatomical location of the tumor within the colon [29].

**Figure 2:**
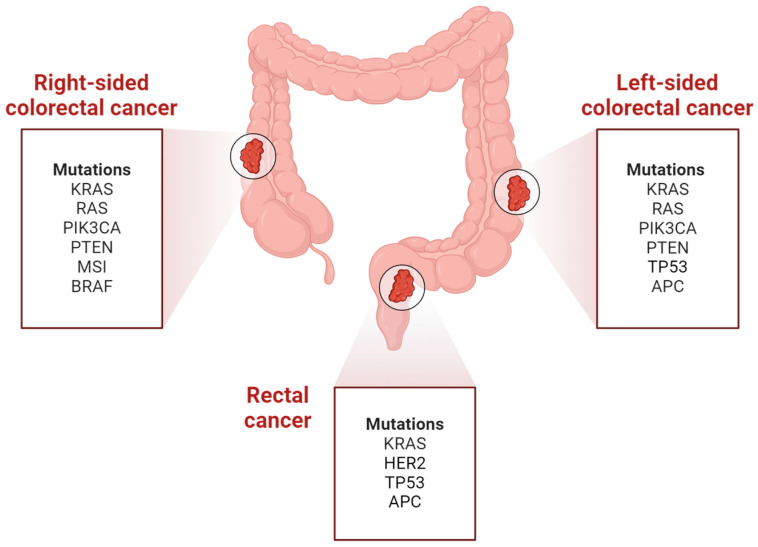
Molecular biology of colorectal cancer stratified by tumor location.

Data from The Cancer Genome Atlas has shown a variation in the distribution of molecular subtypes between right- and left-sided tumors [Figure 2]. However, these findings are not uniform and present some inconsistencies. For example, there is evidence indicating a more favorable prognosis for left-sided tumors compared to right-sided ones irrespective of RAS mutational status [27,30]. Moreover, tumors characterized by a higher mutation burden, often defined as having more than 10 mutations per megabase, and exhibiting microsatellite instability-high (MSI-H) status, associated with a more favorable outcome, are significantly more prevalent in the right-sided colon [31,32].

This indicates that while there is a recognizable difference in the molecular characteristics of tumors based on their location in the colon, the specifics of these differences and their implications are not entirely clear or consistent across different studies.

### 3.3. Local Tumor Extent

The local extent of disease, specifically the depth of tumor penetration, is an independent factor that impacts survival [33,34]. However, the assessment and reporting of features determining the T category, especially the presence or absence of serosal involvement, exhibit variability. There is notable confusion surrounding the definition of serosal “involvement”. Histologically determining serosal penetration can be challenging, and adopting a conservative interpretation may result in an understaging of the disease. For instance, in some cases, cytologic examination of serosal scrapings has identified malignant cells in as much as 26% of specimens histologically defined pT3 [35,36].

When there is uncertainty regarding the maximum extent of tumor penetration, the American Joint Committee on Cancer (AJCC) staging guidelines justify assigning the lesser value. The AJCC Cancer Staging Manual recommends employing multiple-level sectioning and/or submitting additional tissue blocks to thoroughly evaluate serosal involvement. This involvement, whether due to direct tumor extension, or due to inflammation, is classified as T4a in the staging system [37]. This approach is designed to ensure more accurate and precise staging, particularly in complex or borderline cases.

The prognostic significance of a tumor that closely approaches the serosal surface (within less than 1 mm) but does not penetrate it remains unclear. This situation may indicate a higher risk of peritoneal relapse. The implications of such proximity to the serosal surface without actual penetration is an area of ongoing research, and further studies are needed to establish the prognostic inference and guide clinical decision-making in these cases [38].

The histopathologic characteristics of tumoral involvement of the peritoneum are diverse and varied. This heterogeneity presents a challenge in both diagnosis and interpretation. Currently, there is a lack of standardization for the diagnostic interpretation of these histopathologic findings. This gap in standardization can lead to variability in diagnosis and treatment decisions. Local peritoneal involvement includes any of the following: 1. a mesothelial inflammatory or hyperplastic reaction with the tumor close to the serosal surface; 2. tumor presence at the serosal surface with an inflammatory reaction; 3. free tumor cells within the peritoneum, associated with ulceration. All three types constitute a serosal involvement-T4a tumor [38]. However, the presence of free tumor cells on the serosal surface is more likely to be associated with intraperitoneal recurrence [35].

### 3.4. Regional Lymphatic Nodes

Regional lymph node involvement stands out as one of the most significant predictors of postoperative outcome for colorectal cancer (CRC), second only to the presence of distant metastasis. The spread of cancer to the lymph nodes is a crucial factor guiding the recommendation for adjuvant therapy in both colon cancer and rectal cancer. The relationship between lymph node metastases and the development of distant metastases in CRC is a subject of ongoing debate. There are two contrasting theories: one proposes that lymph node metastases may serve as a source of distant metastatic seeding, while the other suggests that regional lymph nodes act as a barrier, trapping primary CRC cells that inherently possess the potential to metastasize [39].

The mechanism of distant metastatic spread is generally believed to follow venous rather than lymphatic drainage pathways. Supporting this view, some data indicate that in a significant number of cases, lymphatic metastases and distant metastases originate from independent tumor subclones [40,41]. The likelihood of regional lymph node involvement is closely linked to two key factors: the depth of the primary tumor’s transmural invasion and the histologic grade of the tumor. Additionally, the number of lymph nodes involved is a significant predictor of the patient’s prognosis [42,43,44]. The overall lymph node count within surgical specimens plays a crucial role in determining the prognosis for both stage II (node-negative) and stage III (node-positive) colorectal cancer [45,46,47,48].

The lymph node ratio (LNR), the ratio of metastatic lymph nodes to the total number of lymph nodes examined, has been proposed as a method for enhancing prognostic stratification in colorectal cancer. The LNR offers a more nuanced assessment of lymph node involvement, potentially providing more accurate prediction of patient outcomes [49,50]. For example, the INT-0089 trial in which the main focus was to determine the relationship between overall survival and the number of lymph nodes analyzed from CRC surgical specimens, has reported the number of analyzed lymph nodes itself as an independent prognostic variable [51].

The association between the total number of lymph nodes in the surgical specimen and patient outcomes is not entirely clear, and while increased accuracy of staging due to the removal of more nodes is a plausible explanation, it does not fully account for the strong association with survival [52,53].

One possibility is that higher total lymph node count may indicate a more meticulous surgical approach. Additionally, a robust immune response, reflected in a higher lymph node count, might play a role in controlling or eliminating residual cancer cells, impacting survival.

Tumors with a more aggressive behavior might have a higher likelihood of metastasizing to lymph nodes, leading to a higher total node count and worse outcomes.

Despite variations in research findings, guidelines from expert groups advise histological examination of at least 12 lymph nodes to accurately determine nodal status in colorectal cancer. It is important to note that this recommendation was derived empirically from older observational data and may not have accounted for variables such as T-stage, tumor grade, and the use of preoperative chemoradiotherapy for rectal cancer [45,46,54,55]. The number of nodes contained in a colorectal cancer resection specimen can vary due to surgical technique. Factors such as the surgeon’s approach, thoroughness in searching for nodes, and the use of techniques like fat clearing to enhance macroscopic visualization of nodes may contribute to these variations [56]. When the number of nodes present in the surgical specimen is fewer than 12, guidelines suggest employing additional techniques, such as fat clearing, to enhance nodal yield [57]. This approach aims to improve the accuracy of staging by ensuring a more comprehensive evaluation of lymph nodes, crucial for appropriate treatment planning and prognostic assessment in CRC.

Relevance of the traditional 12-node threshold has been put into question, particularly in patients with rectal cancer who undergo neoadjuvant therapy before resection. In this context, achieving the removal of 12 or more nodes may often be challenging. Importantly, lower nodal counts following neoadjuvant therapy have not consistently been linked to understaging or inferior survival outcomes. The impact of neoadjuvant therapy on lymph nodes, including changes in nodal architecture and potential tumor downsizing, can influence the number of nodes retrieved during surgery. Therefore, some studies and experts argue that rigid adherence to the 12-node threshold may not be as critical in cases involving preoperative treatment for rectal cancer [58].

There is ongoing debate regarding the prognostic importance of isolated tumor cells (ITCs) and micrometastases in regional lymph nodes. Some authors define ITCs as clumps of up to 20 tumor cells measuring ≤0.2 mm [59]. Micrometastases are defined as clusters of tumor cells measuring ≥0.2 mm. The detection and significance of these minute cancer cell presences have gained prominence due to advancements in sentinel node mapping and sensitive detection techniques [60,61].

Sentinel node mapping, a procedure used to identify the first lymph node(s) that a cancer is likely to spread to from the primary tumor, has been instrumental in focusing attention on the role of micrometastases and ITCs. Additionally, techniques like immunohistochemistry (IHC) and reverse-transcriptase polymerase chain reaction (RT-PCR) have enhanced the ability to detect very small clusters or even single tumor cells in lymph nodes. These methods are more sensitive than traditional histopathological examination and can identify tumor-specific RNA, providing a more detailed analysis of lymph node involvement. Several studies suggest that the use of these supplemental assays (lymph node ultrastaging) could result in upstaging of up to 50% of CRC patients with histologically negative nodes [62,63,64]. Sloothaak et al. [65] reported in a meta-analysis that patients with micrometastases had a poor prognosis, whereas those with ITCs did not. Based on this meta-analysis, the eighth edition of the AJCC/UICC TNM staging system classifies nodal micrometastases as pN+, while ITCs are classified as pN0.

### 3.5. Lymphovascular and Perineural Invasion

Tumor invasion into veins or small nonmuscularized vessels, such as postcapillary lymphatics or venules, is a significant factor in determining prognosis. This type of invasion indicates a more advanced stage of cancer, suggesting the tumor’s ability to spread beyond its original site through the vascular or lymphatic systems. The presence of such invasion often implies a higher risk of metastasis and recurrence, influencing treatment decisions and overall patient management [8,66,67].

Due to its prognostic significance, lymphovascular invasion (LVI) is included as a clinicopathologic factor in the definition of “high-risk” stage II colon cancer by the American Society of Clinical Oncology (ASCO), National Comprehensive Cancer Network (NCCN), and European Society for Medical Oncology (ESMO). Similarly, there is level 1 evidence that perineural invasion (PNI) is associated with poor prognosis in multivariate analysis [68,69,70] and, like LVI, is included in the definition of “high-risk” stage II colon cancer.

### 3.6. Histologic Type, Grade of Differentiation, and Presence of Mucin

In general, histologic type is not established as an independent prognostic factor for colorectal adenocarcinomas, except for certain high-grade subtypes like signet ring, poorly differentiated, or undifferentiated tumors [71,72]. However, histologic grade, which indicates the extent of tumor differentiation, consistently serves as a prognostic factor regardless of the cancer stage [73,74]. The histologic grade of differentiation considers the presence of well-formed glands, which indicate the extent of cell–cell cooperation, maintenance of polarization, and coordinated secretion of cell products into a central lumen. Higher levels of these features correspond to greater differentiation and a lower grade. The incorporation of cytologic or other characteristics in grade estimation varies. Well-differentiated and moderately differentiated tumors (classified as low-grade in a two-tiered system) typically exhibit varying degrees of gland formation. In contrast, poorly differentiated or undifferentiated adenocarcinomas (classified as high-grade tumors) lack well-defined glandular structures and primarily consist of solid sheets or cords of infiltrating cells, often accompanied by marked cellular atypia, pleomorphism, and a high mitotic rate. Nevertheless, the subjective nature of histologic grading introduces notable interobserver variability, exacerbated by the lack of a universally accepted and consistently applied grading system [75,76].

Numerous colorectal tumors generate mucin, which can either be retained within the cells (signet ring cells) or secreted. When extracellular mucin is present, it can permeate the colonic wall, facilitating local tumor spread [77]. Tumors that produce substantial amounts of extracellular mucin, constituting at least 50 percent of the tumor mass, are categorized as mucinous carcinomas. Although signet ring cancers are unequivocally linked to a poorer prognosis, the impact of an extracellular mucinous component on prognosis remains uncertain, as the existing data are contradictory [72]. Several studies indicate that the presence of mucin is independently linked to poorer outcomes in rectal tumors, but not necessarily in colonic tumors [72]. However, conflicting data exist regarding the independent prognostic impact of the mucinous subtype in rectal cancer [78]. Some studies suggest modern treatment approaches, such as preoperative short-course radiation therapy or long-course chemoradiotherapy for rectal cancer, may have contributed to improved outcomes, narrowing the gap between common adenocarcinomas and mucinous cancers [79].

The two main types of mucins associated with CRC are MUC2 and MUC5AC, although others may also be implicated. MUC2 is a gel-forming mucin primarily produced by goblet cells in the colon and is responsible for maintaining the protective mucus barrier in the gastrointestinal tract. In CRC, alterations in MUC2 expression and glycosylation have been observed. Aberrant MUC2 expression, such as reduced or absent expression, is associated with tumor progression, invasion, and metastasis. Moreover, changes in MUC2 glycosylation patterns, including truncated or aberrantly glycosylated forms, have been linked to CRC aggressiveness and poor prognosis. These alterations in MUC2 expression and glycosylation contribute to disrupted mucin barrier function and promote tumor cell invasion and metastasis [80,81,82].

MUC5AC is a mucin glycoprotein primarily expressed by surface epithelial cells in the gastrointestinal tract. In CRC, its upregulation is particularly evident in mucinous adenocarcinomas and is associated with tumor aggressiveness, poor differentiation, and adverse clinical outcomes [83]. MUC5AC expression is linked to the serrated neoplasia pathway, characterized by CpG island methylator phenotype (CIMP) positivity, BRAF p.V600E mutations, mismatch repair deficiency, and advanced tumor stage. It is more frequently observed in ulcerative colitis-associated cancers and correlates with poor prognosis, lymphovascular invasion, metastasis, and right-sided colon predominance. Furthermore, its presence in patient sera is tied to reduced survival [83]. Despite these data that suggests that MUC5AC is associated with an unfavorable prognosis in CRC patients, another study [84] reports that tumor location and dMMR independently predict MUC5AC expression, and that it does not correlate with markers of aggressiveness, such as tumor invasion (pT) or nodal involvement (pN), in dMMR or proficient MMR (pMMR) cases.

Despite in vitro evidence suggesting a role in tumorigenesis, its clinical prognostic impact remains inconsistent. However, MUC5AC shows promise as a therapeutic target, exemplified by treatments like Ensituximab, indicating potential utility in managing dMMR CRC cases.

Besides MUC2 and MUC5AC, other mucins, such as MUC1, MUC4, and MUC6, may also be dysregulated in CRC and contribute to tumor progression. For example, aberrant expression of MUC1, a transmembrane mucin, has been associated with CRC metastasis and poor prognosis. Similarly, altered expression of MUC4 and MUC6 has been reported in CRC and may influence tumor aggressiveness and patient outcomes. However, the specific roles of these mucins in CRC pathogenesis and their clinical implications require further investigation [85].

Data suggest colorectal tumors linked to MMR (mismatch repair) deficiency tend to have a more favorable prognosis regardless of mucin secretion or mucinous subtype. This observation was demonstrated in a series of 394 consecutive patients with stage III colorectal cancer who underwent adjuvant chemotherapy; the three-year survival rate was significantly higher for those with nonmucinous histology (79% versus 57%). Notably, among the 26 patients with tumors deficient in one or more MMR proteins, there was no discernible difference in outcomes between mucinous and nonmucinous tumors [86].

### 3.7. Tumor Budding

“Tumor budding” is a distinctive feature of the tumor border, characterized by microscopic clusters of undifferentiated cancer cells located just ahead of the invasive front of the tumor. This phenomenon is also described as “focal dedifferentiation” [87]. It is suggested that tumor budding represents the detachment of cells and invasion into the stroma at the leading edge of a carcinoma, likely an early stage in the metastatic process. Certain data propose that tumor budding, particularly when it is extensive, might hold greater prognostic significance compared to tumor grade. Furthermore, some evidence suggests that the prognostic value of tumor budding is independent of the overall configuration of the tumor border [87,88]. The International Tumor Budding Consensus Conference has endorsed a three-tiered grading system for tumor budding (BD1 through 3), where specimens with BD3 budding (≥10 buds) are associated with higher risk of recurrence in stage II colorectal cancer [89]. A systematic review has further concluded that resected colorectal cancers with high levels of tumor budding are more likely to experience disease recurrence (odds ratio 5.50, 95% CI 3.64–8.29) and cancer-related death at five years (odds ratio 4.51, 95% CI 2.55–7.99) compared to those with lower levels of tumor budding [90].

High levels of tumor budding are included among the clinicopathologic features of high-risk stage II colon cancer, aiding in the decision-making process for adjuvant chemotherapy. According to an updated ASCO (American Society of Clinical Oncology) guideline on the treatment of stage II cancer, patients whose tumors exhibit this feature may be offered systemic therapy [91].

## 4. Molecular Factors

### 4.1. RAS and BRAF Mutation

**Figure 3:**
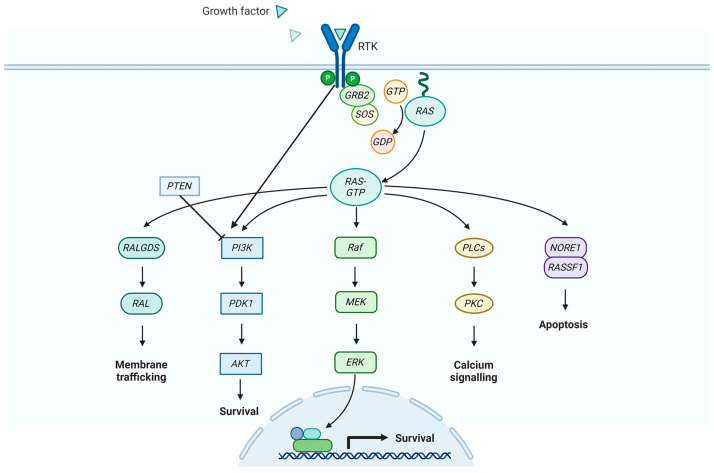
RAS/BRAF/MEK/MAPK and PI3K/PTEN/AKT signaling pathways. The PI3K/AKT/mTOR and RAS/RAF/MEK/MAPK pathways are key signaling routes activated by growth factor receptors like HER2, EGFR, and IGF-1R.

The development of CRC entails the buildup of genetic and epigenetic alterations within pathways governing cell proliferation, apoptosis, and angiogenesis. Mutations involving RAS and BRAF hold prognostic and predictive significance in metastatic colorectal cancer. Most of the research into predictive and prognostic biomarkers has traditionally concentrated on assessing EGFR expression and subsequently examining changes in the RAS/BRAF/MEK/MAPK and PI3K/PTEN/AKT pathways [Figure 3].

Clinical trial data reveal RAS mutations to be predictive of unresponsiveness to monoclonal antibodies targeting EGFR (panitumumab/cetuximab) across all treatment lines, potentially even worsening outcomes when combined with oxaliplatin-based chemotherapy [92,93,94]. While chemotherapy plus bevacizumab is a standard first-line treatment for these patients, it has its limitations. Notably, RAS mutations may lead to reduced benefits from chemotherapy in addition to bevacizumab compared to RAS wild-type mCRC [95,96]. Despite these findings, data from the JACCRO CC-11 trial suggests that first-line mFOLFOXIRI (leucovorin, 5-FU, oxaliplatin, irinotecan) with bevacizumab is effective in patients with RAS-mutated mCRC [97]. Potentially effective second-line therapies for patients diagnosed with RAS-mutated mCRC include FOLFIRI in combination with aflibercept or ramucirumab [98]. A retrospective study including 151 patients with mCRC that received double doses of bevacizumab (i.e., 10 mg/kg of body weight) after first progression on a standard dose of bevacizumab (i.e., 5 mg/kg of body weight) did, however, report that increasing bevacizumab dose could have a favorable prognostic impact [99].

Testing for RAS mutational status is advisable for all patients diagnosed with metastatic colorectal cancer (mCRC). The phase III PRIME study highlighted the importance of analyzing KRAS mutations in exons 2, 3, and 4, as well as NRAS mutations in exons 2, 3, and 4 [93]. A next-generation sequencing (NGS)-expanded RAS panel, utilizing formalin-fixed paraffin-embedded tumor samples, has recently been validated for clinical use in patients with metastatic colorectal cancer [100]. The current therapeutic landscape of mCRC necessitates precise identification of RAS mutations, as evidenced by the approval of KRAS G12C selective inhibitors sotorasib and adagrasib. The phase I CodeBreaK 100 trial [101] investigated the safety and antitumor effects of sotorasib in patients with heavily treated KRASG12C tumors, which included a subset of 42 patients with previously treated mCRC. Among this subgroup, 3 patients (7.1%) achieved a partial response (PR), while 31 patients (71.8%) experienced stable disease (SD) as their best response. The median progression-free survival (mPFS) was 4.0 months (ranging from 0.0 to 11.1 months). The treatment was generally feasible, with diarrhea, fatigue, and nausea being the most commonly reported adverse events.

In a subsequent phase II expansion cohort [102], the clinical efficacy of sotorasib was assessed in 62 patients with refractory KRASG12C-mutant mCRC. Out of these patients, 6 (10%) achieved a partial response, and 45 (73%) experienced stable disease. The mPFS was 4 months (with a 95% confidence interval ranging from 2.8 to 4.2 months), and median overall survival (mOS) was 10.6 months (with a 95% confidence interval ranging from 7.7 to 15.6 months).

In the KRYSTAL-1 trial [103], which explored adagrasib either as a standalone treatment or combined with cetuximab, findings revealed that among 43 patients administered adagrasib alone, overall response rate (ORR) was 19% (8 out of 43), accompanied by a notably high disease control rate (DCR) of 86% (37 out of 43). The median progression-free survival (mPFS) was 5.6 months (with a 95% confidence interval of 4.1 to 8.3 months), and median overall survival (mOS) was 19.8 months (with a 95% confidence interval of 12.5 to 23 months). In the same study, for the 28 patients treated with cetuximab in combination with adagrasib, the overall response rate (ORR) was 46% (13 out of 28), with a disease control rate (DCR) of 100% (28 out of 28). Median progression-free survival (mPFS) was 6.9 months (with a 95% confidence interval of 5.4 to 8.1 months), and median overall survival (mOS) was 13.4 months (with a 95% confidence interval of 9.5 to 20.1 months).

It is important to note, however, that the 12-month overall survival (OS) rate was similar between monotherapy and combination treatment, and the mOS might be influenced by the short follow-up duration and limited number of patients in the study.

Clinical trial data indicate that the presence of mutated BRAF V600E serves as a negative prognostic indicator for patients diagnosed with metastatic colorectal cancer (mCRC), potentially indicating resistance to EGFR-antibody therapy, particularly in heavily treated individuals [104,105,106]. Even so, the predictive significance of BRAF V600E mutations in earlier lines of therapy remains uncertain. A meta-analysis of randomized controlled trials suggested insufficient evidence to support the notion that BRAF V600E mutations are a negative predictive marker for response to EGFR inhibitors [107].

Another meta-analysis [108] demonstrated that anti-EGFR treatment did not improve progression-free survival (PFS) or overall response rate (ORR) in patients with BRAF-mutated mCRC. The phase II VOLFI trial [109] reported that the addition of panitumumab to mFOLFOXIRI among RAS wt patients improved the ORR and rate of secondary metastasectomy. A subgroup analysis found an increased ORR among BRAF-mutated patients, although no differences were reported in mPFS between the two arms. A more recent phase III trial [110] concluded that increasing the potency of initial chemotherapy alongside panitumumab in molecularly chosen, predominantly left-sided mCRC patients does not yield treatment advantages but does lead to a notable rise in gastrointestinal side effects. Therefore, triplet chemotherapy and EGFR antibodies should not be used as first-line therapy among mCRC, irrespective of BRAF mutational status.

Current therapeutic approaches involve combining chemotherapy with anti-VEGF antibodies. A subset analysis of the TRIBE study [101] and findings from other patient cohorts suggest that FOLFOXIRI plus bevacizumab could be advantageous as a first-line treatment for these patients, as recommended by ESMO guidelines for those with BRAF-mutated mCRC. Additionally, other agents targeting vascular endothelial growth factor (VEGF) may also demonstrate efficacy in this patient subgroup. An interesting aspect of mCRC treatment is that BRAFmt patients seem to exhibit a superior response to VEGF targeting agents. This was reported in the VELOUR [111] clinical trial in which the advantages of combining aflibercept with FOLFIRI in patients with mCRC previously treated with oxaliplatin remained consistent across various patient subgroups, including those who had received prior bevacizumab treatment and those who had not. In a subgroup analysis of the VELOUR study, BRAFmt mCRC patients exhibited a larger, although non statistically significant, benefit in mOS [112].

As monotherapy with BRAF V600E inhibitors has been shown to cause activation of EGFR, which in turn causes tumor proliferation, current therapeutic options associate BRAF inhibitors with EGFR antibodies. In the BEACON mCRC study [113], the combination of encorafenib and cetuximab demonstrated improved mOS, ORR, and mPFS compared to standard chemotherapy in patients previously treated for metastatic colorectal cancer. Similarly, the SWOG S1406 trial reported that concurrently targeting EGFR and BRAF, in conjunction with irinotecan, is effective in treating mCRC with the BRAFV600E mutation [114].

While the predictive and prognostic significance of KRAS and BRAF mutations in metastatic colorectal cancer (mCRC) is well established, their impact on localized colorectal cancer (CRC) remains not fully elucidated. A recent pooled analysis of seven clinical trials [115] has reported that KRAS exon 2 and BRAFV600E mutations correlate with a shorter time to recurrence (TTR) in patients with surgically removed stage III microsatellite stable (MSS) colon cancer, whereas TTR remains unaffected in patients with microsatellite instability-high (MSI-H) tumors. BRAFV600E, KRAS G12C, and G13D mutations are linked to shorter survival after recurrence (SAR) in patients who underwent surgery for stage III microsatellite stable (MSS) colon cancer. This analysis concluded that testing for both KRAS and BRAFV600E mutations in stage III patients is warranted as it can provide a more precise definition of individual patient prognosis. Additionally, such testing may facilitate patient selection for (neo)adjuvant trials targeting specific molecular subtypes associated with poorer prognosis.

### 4.2. Mismatch Repair Deficiency

Mutations in one of several DNA MMR genes are prevalent in Lynch syndrome, also known as hereditary nonpolyposis colorectal cancer (HNPCC), as well as in 15 to 20 percent of sporadic colon cancers [116]. The distinctive genetic profile of dMMR tumors involves a substantial count of DNA replication errors (known as RER+) and elevated levels of DNA microsatellite instability (MSI), typically defined as instability present in 30 percent or more of microsatellite loci [117,118]. Microsatellite instability (MSI) refers to the variation in the number of repeated DNA sequences, resulting from the insertion or deletion of these repeated units, due to MMR deficiency.

In localized colorectal cancers, tumors characterized as dMMR (MSI-H) exhibit longer survival rates compared to proficient MMR (pMMR) tumors. These pMMR tumors may either display low microsatellite instability (MSI-L), indicating instability in less than 30 to 40 percent of microsatellite loci, or may be microsatellite stable (MSS). This survival advantage is observed in both Lynch-related and sporadic cases, despite dMMR tumors often being poorly differentiated [119,120,121,122].

Data from the PETACC-3 trial indicate that tumor specimens identified as MSI-H are more prevalent in stage II disease compared to stage III disease (22% vs. 12%, respectively; *p* < 0.0001) [123]. In another extensive study, the proportion of stage IV tumors characterized as MSI-H was only 3.5% [124]. These findings imply that MSI-H (i.e., dMMR) tumors have a reduced propensity for metastasis. Indeed, considerable evidence suggests that in patients with stage II disease, a deficiency in MMR protein expression or MSI-H tumor status serves as a prognostic indicator for a more favorable outcome [125,126,127]. In contrast, the beneficial impact of dMMR on outcomes appears to be more limited in stage III colon cancer and may vary depending on primary tumor location [121,125].

Some of these aforementioned studies suggest that a deficiency in MMR protein expression or MSI-H tumor status may predict reduced benefit and potentially even detrimental impact of adjuvant therapy with a fluoropyrimidine alone in patients with stage II disease [120,126,127,128]. A retrospective study involving long-term follow-up of patients with stage II and III disease, stratified by MSI tumor status, demonstrated that those characterized as MSI-L or MSS showed improved outcomes with 5-FU adjuvant therapy. Patients with tumors characterized as MSI-H did not exhibit a statistically significant benefit from 5-FU after surgery, instead demonstrating a lower 5-year survival rate compared to those who underwent surgery alone [126]. Similarly, findings from another retrospective study pooling data from adjuvant trials by Sargent et al. [128] showed that in tumors characterized as dMMR, adjuvant 5-FU chemotherapy appeared to be detrimental in patients with stage II disease but not in those with stage III disease.

In contrast to the findings of Sargent et al., a paper involving 1,913 patients with stage II colorectal cancer from the QUASAR study, half of whom received adjuvant chemotherapy, revealed that although dMMR was prognostic (with a recurrence rate of 11% for dMMR tumors vs. 26% for pMMR tumors), it did not predict the beneficial or detrimental impact of chemotherapy [120]. A study of patients from the CALGB 9581 and 89803 trials arrived at similar conclusions [129]. MMR status was prognostic but not predictive of the beneficial or detrimental impact of adjuvant therapy (specifically, irinotecan plus bolus 5-FU/LV [IFL regimen]) in patients with stage II colon cancer.

In the metastatic setting, MSI-H tumors are linked to a poor prognosis, though this may be in part due to a higher prevalence of BRAF mutations in patients with MSI-H tumors compared to those with proficient mismatch repair (pMMR) (*p* < 0.001) [130]. While some studies suggest that MSI status does not predict the efficacy of chemotherapy or targeted therapy in mCRC [131,132], a recent randomized phase III trial found that patients with MSI-H tumors experienced a longer overall survival when treated with chemotherapy plus bevacizumab compared to cetuximab (*p* < 0.001) [133].

dMMR tumors harbor numerous mutations, leading to the production of mutant proteins that have the potential to be recognized and targeted by the immune system. However, tumor cells produce programmed death-ligands 1 and 2 (PD-L1 and PD-L2), which can suppress the immune response by binding to the programmed cell death protein 1 (PD-1) receptor on T-effector cells. This mechanism evolved to protect the host from an uncontrolled immune response, but many tumors evade immune surveillance by increasing PD-L1 expression. Consequently, it was proposed that dMMR tumors might respond to PD-1 inhibitors. Several clinical trials such as KEYNOTE-177 [134] and CheckMate-142 [135] have led to the approval of Pembrolizumab and Nivolumab as first line therapies in MSI-H/dMMR mCRC. A recent phase III trial (CheckMate-8HW) [136] in which nivolumab plus ipilimumab was evaluated as a first-line treatment option for MSI-H/dMMR mCRC has been shown to significantly prolong progression-free survival (PFS) compared to standard chemotherapy with or without the addition of targeted therapy. This outcome successfully met the primary endpoint of the study, underscoring the efficacy of the dual immunotherapy regimen as a potential treatment option for first-line metastatic colorectal cancer (mCRC).

### 4.3. HER2 Amplification or Mutation

**Figure 4:**
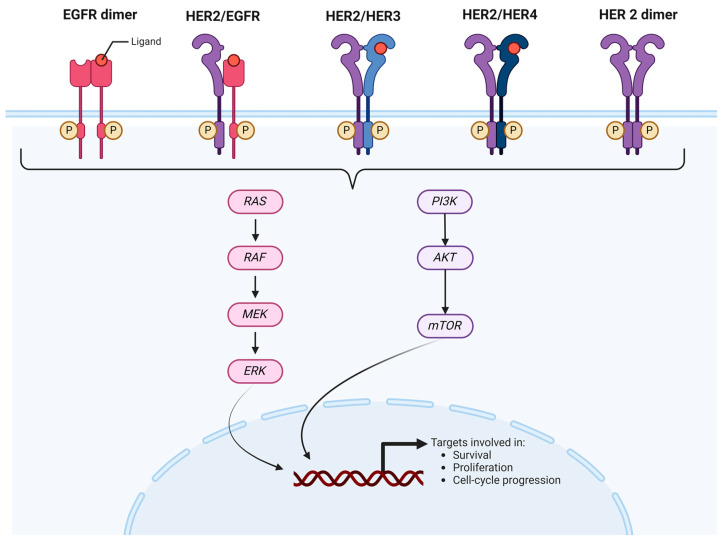
HER2 signaling, where ligand binding or dimerization activates pathways like RAS-RAF-MEK-ERK and PI3K-AKT-mTOR, driving proliferation, survival, and cell-cycle progression.

The erb-B2 receptor tyrosine kinase 2 gene, commonly known as HER2, is a proto-oncogene situated on chromosome 17q21. It encodes a transmembrane glycoprotein receptor with tyrosine kinase activity. Unlike other members of the EGFR family, HER2 does not directly bind ligands. However, its homodimerization or heterodimerization with other EGFR family members (such as HER1/EGFR, HER3, HER4) triggers the transphosphorylation of the intracytoplasmic tyrosine kinase domain, activating various downstream signal transduction pathways (such as RAS/RAF/ERK, PIK3K/AKT/mTOR) [Figure 4]. 

Amplification of the HER2 oncogene or overexpression of its protein leads to heightened mitogenic signals, even without ligands bound to other receptors. This hyperactivation results in uncontrolled cell proliferation and tumorigenesis [137,138,139,140].

The significance of HER2 as a biomarker indicating poor prognosis in breast and gastric cancer is widely recognized. Amplification of the HER2 gene or overexpression of its protein has been effectively targeted by a range of agents, including trastuzumab, pertuzumab, lapatinib, neratinib, trastuzumab emtansine, tucatinib, trastuzumab deruxtecan, and margetuximab [141,142,143]. Initially, HER2 was identified as a negative predictive biomarker in metastatic colorectal cancer (mCRC), its amplification or overexpression correlating with resistance to anti-EGFR treatments. Recognizing this, several trials investigated HER2 as a potential novel therapeutic target in mCRC, evaluating the effectiveness of anti-HER2 agents in this small, molecularly selected population.

A phase II study by Ramanathan et al. [144] evaluated the role of trastuzumab and irinotecan as first or second line therapies in advanced CRC. Only seven patients were included in this cohort, five (71%) demonstrated an objective response, with these responses being sustained for at least 6 months. However, due to insufficient patient enrollment, the study was prematurely terminated. Another phase II trial [145] evaluated the combination of trastuzumab plus FOLFOX (leucovorin calcium, fluorouracil, oxaliplatin) in patients with HER2-positive (IHC 2+ or IHC 3+) metastatic colorectal cancer as second- or third-line therapy. Among the 21 patients included in this cohort, five (24%) achieved either a complete response or a partial response, with a median duration of response of 4.5 months. However, this study was discontinued prematurely due to lack of efficacy.

Several other phase II trials evaluated the role of HER2 dual blocade, the HERACLES studies included most of these trials. The objective of HERACLES cohort A [146] was to examine the efficacy of dual HER2 blockade with trastuzumab plus lapatinib in patients with KRAS exon 2 wild-type metastatic colorectal cancer (mCRC) who exhibited HER2 amplifications and/or HER2 overexpression. A total of 914 patients with disease refractory to standard treatments underwent screening, among whom 48 (5%) were found to have HER2-positive tumors. Out of the 27 patients enrolled in the trial, eight (30%) achieved an objective response. Among them, seven patients (26%) experienced a partial response, while one patient (4%) maintained a complete response for over 7 years. Additionally, 12 patients (44%) showed stable disease as their best response, resulting in a disease control rate of 59%. The median duration of response was 38 weeks, with a median progression-free survival (PFS) of 5.3 months and a median overall survival (OS) of 11.5 months. Notably, seven out of eight responders (88%) had a HER2 score of 3+ in immunohistochemistry (IHC), whereas only 10 out of 17 non-responders (59%) had the same score. This suggests that patients with higher HER2 amplification levels may derive greater benefit from the treatment.

In the HERACLES cohort B, a single-arm phase II trial, the combination of pertuzumab and the antibody-drug conjugate trastuzumab emtansine was investigated in patients with histologically confirmed RAS/BRAF wild-type HER2-positive metastatic colorectal cancer (mCRC) refractory to standard treatments. The primary endpoint, set at achieving an objective response rate (ORR) of at least 30%, was not met, with only three patients (9.7%) achieving an objective response [147].

In the MyPathway trial [148], a phase II basket trial spanning various solid tumors, patients diagnosed with HER2-positive tumors received a combination treatment of trastuzumab and pertuzumab. Among the 57 enrolled patients with HER2-amplified metastatic colorectal cancer (mCRC), one individual (2%) achieved a complete response, while 17 patients (30%) saw partial responses. Consequently, 18 out of the 57 patients (32%) achieved an objective response (95% CI, 20–45), with four patients maintaining a response duration exceeding 12 months. An interesting aspect of this study is that in a posthoc analysis out of 13 patients with KRAS mutations present only one exhibited an objective response which is much lower than the KRASwt cohort, moreover shorter mPFS and mOS were observed in the KRASmt group.

The TAPUR Study is a phase II basket trial [149] aimed at assessing the antitumor efficacy of commercially available targeted agents in patients with advanced cancers exhibiting potentially actionable genomic alterations. The study data include two cohorts focusing on patients with colorectal cancer (CRC), one with ERBB2 amplifications and the other with either ERBB2 or ERBB3 (ERBB2/3) mutations, who received treatment with pertuzumab plus trastuzumab. In the study, a cohort of 28 patients diagnosed with HER2 amplification participated. The outcomes of this trial are regarded as favorable for HER2-amplified tumors, with a disease control rate (DC rate) in this subgroup calculated at 54%. The median progression-free survival (mPFS) stood at 17.2 weeks, while the median overall survival (mOS) reached 60 weeks. An interesting aspect of this study was that in the cohort with either ERBB2 or ERBB3 (ERBB2/3) mutations the DC rate was only 10%, which the trial considered negative. Based on these results it seems that HER2-activating mutations are involved in resistance to anti-EGFR therapy. This is supported by Kavuri et al. in a study [150] which illustrated that introducing specific mutations into the HER2 gene of colon cells led to the overactivation of the HER2 pathway. This overactivation consequently led to resistance to cetuximab and panitumumab. Similarly, a study by Raghav et al. [151] suggests that HER2 amplification observed in RAS/BRAF wild-type mCRC appears to serve as a predictive biomarker indicating the potential ineffectiveness of anti-EGFR antibody therapy. Moreover, screening patients with RAS/BRAF wild-type mCRC for HER2 amplification is advisable prior to initiating anti-EGFRab treatment. This screening can aid in therapy selection and help identify individuals who may benefit from alternative treatment approaches or early enrollment in clinical trials.

The more recent MOUNTAINEER trial [152], a phase II study, compared the combination of tucatinib plus trastuzumab to tucatinib alone in patients with locally advanced or metastatic colorectal cancer (CRC) refractory to chemotherapy and with wild-type KRAS. The primary endpoint of objective response rate (ORR) was 38.1% for patients receiving tucatinib plus trastuzumab and 3.3% for those receiving tucatinib alone. Disease control was achieved in 16 out of 27 (59%) patients who received the combination therapy. The median progression-free survival (mPFS) and median overall survival (mOS) among patients receiving tucatinib plus trastuzumab were 7 months and 24.1 months, respectively.

The role of anti HER2 ADCs (antibody-drug conjugates) has been assesed in the phase II multicenter DESTINY-CRC01 trial [153]. Patients were administered T-DXd (trastuzumab deruxtecan) at a dosage of 6.4 mg/kg every 3 weeks and were stratified into three cohorts: cohort A (HER2-positive, immunohistochemistry [IHC] 3+ or IHC 2+/in situ hybridization [ISH]+), cohort B (IHC 2+/ISH−), or cohort C (IHC 1+). While patients in cohort A (n = 53) reported an ORR of 45.3%, patients in cohort B or C exhibited no response. A follow-up trial compared the benefit of a reduced T-DXd dose (i.e., 5.4 mg/kg) to the initial 6.4 mg/kg used in cohort A of the DESTINY-CRC01 trial. All patients in this trial were HER2 positive, similar to group A of the DESTINY-CRC01 trial. There was no statistically significant difference between the groups regarding duration of response (5.5 months for both groups) or median progression-free survival (mPFS) (5.8 months for the low-dose group and 5.5 months for the regular-dose group) [154].

### 4.4. POLE/POLD1 Mutations

The genes POLE and POLD1 encode proteins responsible for proofreading during DNA replication, identifying and rectifying mispaired bases. Pathogenic variants (PVs) within the exonuclease domains (ED) of POLE and POLD1 lead to the loss of this proofreading function, causing accumulation of multiple single nucleotide variants (SNVs) over time [155,156].

Zhu et al. [157] substantiated that POLE mutations correlated with a heightened susceptibility to gastrointestinal tumors, suggesting that POLE germline mutation could serve as an effective molecular indicator for prognosticating survival and metastasis. The prevalence of somatic mutations in the exonuclease domain of POLE in colorectal cancer (CRC) is approximately 3%, exceeding that of germline mutations [158].

In the study by Domingo et al. [159], tumors with POLE mutations exhibited higher density of CD8+ lymphocyte infiltration, as determined by immunohistochemistry, compared to overall proficient mismatch repair (pMMR) samples. Interestingly, the number of CD8+ tumor-infiltrating lymphocytes (TILs) did not significantly differ from that observed in deficient mismatch repair (dMMR) tumors. Additionally, there was an elevation in the expression of T lymphocyte markers and effector cytokines, including CD8, Interferon γ, CXCL9, and CXCL10, in POLE-mutated tumors. An increase in the expression of genes encoding immune checkpoint proteins such as PD-1, PD-L1, and CTLA4 was also noted in these tumors.

A retrospective analysis [160] of NGS (next-generation sequencing) data revealed that, among 14,229 reports, 458 POLE mutations were identified (approximately 3.2%), with only 15% of these mutations being pathogenic. Patients treated with PD(L)-1-based immune checkpoint inhibitor (ICI) therapy harboring a pathogenic POLE mutation showed significantly improved clinical benefit rate compared to those with benign variants (82.4% vs. 30%; *p* = 0.013). Moreover, their median progression-free survival (mPFS) and median overall survival (mOS) were notably longer at 15.1 vs. 2.2 months, and 29.5 vs. 6.8 months, respectively, compared to patients with benign variants of POLE mutations. Importantly, in multivariate analysis, the presence of a POLE mutation remained a favorable prognostic factor even after adjusting for microsatellite instability status.

Nevertheless, it is important to note that not all POLE-mutated tumors exhibit a favorable response to checkpoint blockade. For instance, two cases of colorectal cancer with the p.P286R mutation demonstrated either progressive or stable disease after follow-up periods of 1 and more than 10 months, respectively. Both instances exhibited low levels of CD8+ tumor-infiltrating lymphocytes (TILs). In a larger cohort consisting of five microsatellite instability (MSI) tumors and three POLE-mutated tumors, high levels of CD8+ TILs were present in all four patients who responded to treatment, while none of the four patients who did not respond exhibited such levels (*p* = 0.0007) [161].

## 5. Host Immune Response

In various cancer types, the presence of tumor-infiltrating lymphocytes (TILs) has consistently emerged as a positive prognostic indicator across numerous studies. Specifically, a high density of CD8+ T cells and CD45RO+ cells (representing memory CD4+ and CD8+ lymphocytes exposed to antigens) within these TIL populations has been linked to a lack of pathological evidence of early metastatic invasion, earlier disease stage, and improved patient survival. These findings have prompted speculation that this immune response reflects the activation of host defense mechanisms. However, there is a lack of direct evidence to substantiate this hypothesis [162,163,164,165,166].

Evidence indicates that in colorectal cancer, a high density of regulatory T cells, distinguished by the CD4+CD25+ phenotype and believed to regulate the anti-tumor immune response, carries a more robust prognostic significance compared to the infiltration by CD8+ or CD45RO+ cells [167].

Numerous studies on colorectal cancer have highlighted the significance of tumor lymphocytic reaction and T-cell subpopulations as substantial prognostic biomarkers, even following adjustments for stage, lymph node count, and well-established prognostic indicators like BRAF mutation [168,169]. The presence of lymphoid infiltration may serve as a favorable prognostic marker due to its correlation with mismatch repair deficiency (MMR), which manifests biologically as microsatellite instability (MSI). Tumors characterized by abundant lymphoid cells often harbor mutations in DNA MMR genes and exhibit more frequently as MSI-H [170,171,172].

Medullary carcinoma, a distinct histological subtype of colorectal cancer (CRC), typically exhibits microsatellite instability-high (MSI-H) status and is closely linked with Lynch syndrome. It is distinguished by intratumoral lymphocytic infiltrates. Nevertheless, in at least one study, both MSI-H status and intratumoral lymphocytic infiltration were identified as independent prognostic factors [173]. The involvement of tumor infiltrating lymphocytes in the positive prognosis of MSI-H tumors is reinforced by research indicating that certain subgroups of these cells correlate with a unique molecular phenotype [174]. Moreover, the favorable prognosis observed in molecularly defined subcategories of CRC, which are enriched for MSI-H tumors, is marked by heightened expression of genes specific to cytotoxic lymphocytes.

Although compelling evidence supports the significance of the host immune response, it has not yet been established as a standard prognostic indicator for clinical application. However, multinational initiatives are actively underway to integrate tumor and immune factors, aiming to create and validate an “immunoscore”. This score seeks to quantify the in situ immune infiltrate, offering a novel tool for the classification and prognostication of CRC [175,176,177].

The Immunoscore is a prognostic test designed to predict the risk of relapse in individuals with colon cancer by evaluating the host immune response at the tumor site (i.e: TILs) [178,179]. This tool quantifies both CD3+ lymphocytes and CD8+ cytotoxic T cells in the tumor’s center (CT) and invasive margin (IM). Notably, this immune scoring system offers independent and superior prognostic value compared to traditional risk parameters and has been validated by a multinational consortium for stage II and III colon cancer. In early-stage Colon Cancer (CC), a low Immunoscore serves as a dependable marker for identifying patients who are at risk of relapse. This information can prompt healthcare providers to consider implementing a more intensive surveillance program or recommending adjuvant treatment for these individuals [180].

**Figure 5:**
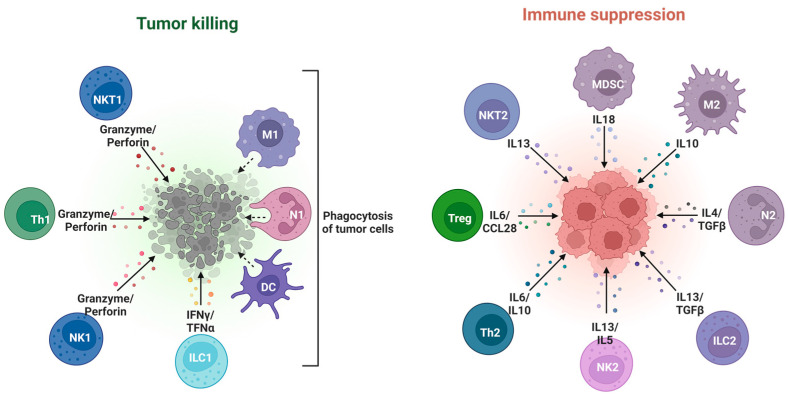
Immune cells in the colorectal microenvironment: On the left, immune cells and their secreted products contribute to an antitumor response, while those on the right are involved in promoting a pro-tumor response.

Numerous investigations have explored the role of inflammatory responses within the tumor microenvironment [Figure 5]. Consequently, an expanding array of inflammatory scores have been proposed to predict survival across various tumor types. Several such inflammatory biomarkers have been integrated into colorectal prognostication models, such as the Glasgow Prognostic Score (GPS) and modified Glasgow Prognostic Score (mGPS) [181]. Additionally, biomarkers like the Neutrophil-to-Lymphocyte Ratio (NLR) [182,183], Platelet-to-Lymphocyte Ratio (PLR) [184], and Lymphocyte-to-Monocyte Ratio (LMR) [185] have undergone extensive research as independent prognostic factors for survival in colorectal cancer patients.

## 6. Predictive Enzymatic Biomarkers in Colorectal Cancer Therapy

Neoadjuvant and adjuvant chemotherapy using fluoropyrimidine-based regimens have shown benefits for numerous patients with CRC, and various markers indicating sensitivity or toxicity to chemotherapy have been suggested.

### 6.1. Dihydropyrimidine Dehydrogenase (DPD)

Dihydropyrimidine dehydrogenase (DPD) is the initial enzyme in the fluoropyrimidine metabolic pathway, playing a crucial role in the breakdown of fluoropyrimidines. The activity of this enzyme can vary significantly, primarily due to genetic variations in the dihydropyrimidine dehydrogenase gene (DPYD). Individuals with partial or complete deficiency in DPD activity may struggle to effectively metabolize fluoropyrimidines, putting them at a higher risk of severe, and potentially fatal, toxicity. Even individuals with partial DPD deficiency face increased risk of serious complications such as severe diarrhea, mucositis, and pancytopenia when administered fluoropyrimidines [186,187,188,189].

Molecular analysis of individuals with DPD deficiency has revealed the presence of over 128 mutations and polymorphisms within the DPYD gene that can lead to either partial or total loss of enzyme activity [190,191,192]. Among them, four mutations have consistently shown a significant correlation with reduced DPD activity and increased susceptibility to fluoropyrimidine toxicity. These mutations include the DPYD2A single-nucleotide polymorphism (SNP) [190,192,193,194,195], DPYD13 SNP [196], DPYD*9B SNP [190,192], and a group of SNPs collectively referred to as HapB3 [195,196,197]. DPYD*2A, classified as a single-nucleotide polymorphism (SNP), corresponds to a splice site variant known as IVS14+1G>A. This variant involves a nucleotide change from guanine (G) to adenine (A) at position 1905+1, leading to the deletion [del] of exon 14. The DPYD*13 SNP involves a nucleotide alteration from thymine (T) to guanine (G) at position 1679, leading to an amino acid substitution where isoleucine (I) is replaced by serine (S) at position 560. DPYD*9B SNP, has a nucleotide change c.2846A>T, with resultant amino acid substitution D949V. HapB3 consists of three intronic variants (c.483+18G>A, c.680+139G>A, and c.959-51T>C), along with one synonymous variant (c.1236G>A). Additionally, there is another intronic variant deep within the DPYD gene, c.1129-5923C>G, which is closely linked with HapB3. This polymorphism, known as c.1129-5923C>G/HapB3, is believed to potentially contribute to the toxicity of fluoropyrimidines. In European populations, c.1129-5923C>G/HapB3 is the most prevalent DPYD variant, with carrier frequencies of around 5% [198]. However, the impact of this variant appears to be modest [199], which could explain the varying reports regarding its influence on fluoropyrimidine toxicity [200].

Although complete DPD deficiency is a rare occurrence, often linked with homozygosity for alleles associated with reduced enzyme activity, partial deficiencies resulting from inheriting one high-risk allele are more prevalent. This trend is particularly notable among black women. An enzyme level analysis conducted on 258 healthy volunteers highlights these demographic disparities [201]. None of the volunteers exhibited complete DPD deficiency. However, partial DPD deficiency was observed in 12.3% of black women, 4.0% of black men, 3.5% of white women, and 1.9% of white men. Only a small fraction (1.6%) of volunteers, comprising three black women and one black man, demonstrated profound DPD deficiency.

The issue of routine DPYD testing remains unclear, with practices differing between the US and Europe. In the US, the prevalence of one of the three risk alleles is generally lower than 10% in most populations, and having a high-risk allele does not always correlate with severe toxicity. In contrast, the Pharmacovigilance Risk Assessment Committee (PRAC) of the European Medicines Agency (EMA) has recommended that all patients planned to receive any fluoropyrimidine therapy undergo pharmacogenomics testing before starting treatment [202]. This approach has been incorporated into updated guidelines for the management of localized colon cancer by the European Society for Medical Oncology (ESMO) [203].

### 6.2. Thymidylate Synthetase Gene (TYMS)

Apart from DPYD, high-risk polymorphisms in the thymidylate synthetase gene (TYMS) might also be linked to a 1.4-to 2.4-fold rise in the risk of severe toxicity from FU-based chemotherapy. Nevertheless, compared to the high-risk DPYD genotypes, the data regarding TYMS are less conclusive.

The expression of TYMS is regulated by transcription factors that bind to its promoter region. Within the 5′ untranslated region (UTR), there are variable numbers of 28 bp tandem repeats (VNTRs), which can enhance transcriptional activity. The majority of individuals carry TYMS alleles containing two or three repeats in this promoter region, labeled as 2R and 3R, respectively [204,205]. Patients who are homozygous for the triple repeat (3R/3R) have an increased number of binding sites for transcription factors and higher TS levels compared to those who are 2R/2R or 3R/2R. Conversely, 2R/2R homozygotes exhibit low TS levels in normal tissues and may be at a higher risk of fluoropyrimidine cytotoxicity. Notably, patients with elevated TS levels demonstrate relative resistance to fluoropyrimidines.

Existing data on the association between these polymorphisms and increased fluoropyrimidine toxicity are contradictory. While the 2R/2R genotype has been linked to greater toxicity in many studies [206,207,208], not all studies have found this association [209]. Even in studies that report a positive association, the sensitivity and positive predictive value seem to be limited. For instance, in three studies involving a total of 200 unselected patients treated with FU, 44 (22 percent) experienced grade 3 or 4 toxicity [205,206,207]. Among these, only 13 had the high-risk 2R/2R genotype (sensitivity 30 percent), and of the 25 patients with the 2R/2R high-risk genotype, only 13 developed grade 3 or 4 toxicity (positive predictive value 52 percent).

### 6.3. UGT1A1 Polymorphisms

Preemptive testing for uridine diphospho-glucuronosyltransferase 1A1 (UGT1A1) genotypes linked to a poor metabolizer phenotype before starting irinotecan remains a topic of debate with evolving perspectives among experts.

SN-38 (active metabolite of irinotecan) undergoes further metabolism mediated by the polymorphic enzyme UGT1A1. Individuals inheriting specific polymorphisms in the UGT1A1 gene or its promoter exhibit reduced enzymatic activity, leading to a “poor metabolizer” phenotype due to diminished clearance of SN-38. This decreased clearance elevates the risk of severe irinotecan-related neutropenia and, to a lesser extent, diarrhea. Most reported data on excess toxicity in these individuals involve those carrying one or more *28 alleles, which represent the most common alteration in European and African ancestries [205]. Additionally, the *6 polymorphism, more prevalent in East Asian ancestry, is linked to irinotecan toxicity. Another relevant polymorphism, *93, associated with heightened SN-38 exposure and irinotecan toxicity, is common in African and European ancestries but less so in East Asian ancestry [210,211].

Some individuals with a poor metabolizer phenotype may be detected due to the presence of Gilbert’s syndrome, an inherited deficiency in UGT1A1 enzyme activity resulting from polymorphisms in the UGT1A1 gene, often involving the *28 allele. Gilbert’s syndrome is characterized by elevated unconjugated bilirubin levels in the blood, typically without symptoms. Otherwise, identifying individuals with a poor metabolizer phenotype necessitates genetic testing for high-risk alleles. In a study involving 1500 patients undergoing routine genotyping at a single institution, 17 percent possessed a UGT1A1 genotype indicative of a poor metabolizer phenotype (*6/*6, *28/*28, *6/*28) [212].

## 7. Other Emerging Biomarkers CRC

Recent studies have revealed several other potentially significant biomarkers, such as Tumor Mutation Burden (TMB), which quantifies the total number of somatic coding errors, base substitutions, and indel mutations present per million bases of DNA. TMB provides an effective estimation of both mutational and neoantigen loads [213,214]. Most tumors with deficient DNA mismatch repair (dMMR) typically exhibit a high Tumor Mutation Burden (TMB), often featuring a median mutation count in the thousands. However, it is important to note that not all tumors with high TMB are dMMR. In instances where tumors have elevated TMB but are not dMMR, the mutation count is considerably lower, approximately 200 nonsynonymous mutations per exome. This translates to around 10 mutations per megabase on the FoundationOne CDx platform [215,216].

With these considerations in mind, TMB has garnered significant attention as a potential biomarker for predicting response to immune checkpoint inhibitor immunotherapy beyond deficient DNA mismatch repair (dMMR). Several retrospective studies conducted across various cancer types have provided evidence supporting the hypothesis that a higher TMB correlates with a more favorable response to immune checkpoint inhibitors [217,218,219]. The fact that a high TMB (hTMB) status identifies a subgroup of patients who may exhibit a robust tumor response to pembrolizumab monotherapy was certified in the KEYNOTE-158 phase II trial [220].

A retrospective study by Wang et al. suggests that a high Tumor Mutation Burden (TMB) indicates a better prognosis in colorectal cancer (CRC) patients with KRAS mutations, thereby confirming the clinical utility of TMB in prognostic assessments [221].

Circulating tumor DNA (ctDNA) represents the portion of circulating DNA originating from a patient’s cancer cells. In colorectal cancer (CRC), tumor cells release DNA into the bloodstream. As detection and quantification techniques for ctDNA have advanced, there has been increased interest in leveraging ctDNA as a highly sensitive marker for assessing the risk of cancer recurrence.

In an initial study involving 27 patients who underwent surgical treatment for colorectal cancer (CRC), circulating tumor DNA (ctDNA) was detected postoperatively in all 14 patients who experienced relapse, whereas none of the non-relapsing patients showed ctDNA presence [222]. Among the 21 patients treated for localized disease, all 6 individuals with ctDNA detected within three months after surgery experienced relapse, in contrast to only 4 out of the remaining 15 patients who showed no ctDNA detection at the three-month mark post-surgery. Notably, those with ctDNA relapse exhibited significantly shorter relapse-free and overall survival compared to those without detectable ctDNA. On average, ctDNA identified relapses after 8.2 months, whereas detection occurred at 12.3 months with carcinoembryonic antigen (CEA) testing, and at 16.9 months with computed tomography (CT) imaging.

In another investigation involving 58 patients who had undergone resection for stage I, II, or III colorectal cancer (CRC), ctDNA was assessed using the Safe-SeqS system one month postoperatively and subsequently at intervals of three to six months [223]. This highly sensitive mutation detection method facilitated the identification of tumoral mutations in the peripheral circulation even at low frequencies. Among those with positive ctDNA, the recurrence rate was 77 percent (10 out of 13), with ctDNA positivity preceding radiographic recurrence by a median of four months (range 2 to 31). Conversely, none of the 45 patients with undetectable ctDNA experienced relapse during a median follow-up of 49 months. Notably, three non-relapsing patients initially showed positive ctDNA results, which later became undetectable with continued follow-up.

In a third investigation, 125 patients diagnosed with stage I, II, or III colorectal cancer (CRC) underwent ctDNA assessment using multiplex, polymerase chain reaction (PCR)-based next-generation sequencing. This approach targeted 16 mutations personalized for each patient, based on whole-exome sequencing of their tumor and matched germline DNA. Evaluations were conducted before surgery, one month postoperatively, and every third month for up to three years [224]. Following definitive therapy, patients testing positive for ctDNA were 40 times more likely to experience disease recurrence (hazard ratio [HR] 43.5, 95% CI 9.8–193.5). Notably, in all multivariate analyses, ctDNA status remained independently associated with relapse after adjusting for known clinicopathologic risk factors. Among the 58 patients who provided post-adjuvant-chemotherapy blood samples, all 7 who tested positive for ctDNA experienced relapse, compared with 7 out of 51 who tested negative (100% versus 14%).

A 2018 joint review by ASCO and the College of American Pathologists (CAP) evaluated the utility of ctDNA analysis in cancer patients. The review concluded that there is currently insufficient evidence of clinical utility and limited evidence of clinical validity for ctDNA assays in early-stage cancer, particularly for post-treatment monitoring or detection of residual disease [68]. There are, however, ongoing efforts to investigate the potential benefits. For instance, a phase II/III trial (NRG GI-005) is planned in North America for patients with resected stage II colon cancer [225]. This trial aims to assess ctDNA clearance with adjuvant chemotherapy and the outcomes for patients with detectable levels of ctDNA receiving adjuvant chemotherapy.

The BESPOKE study [226] aims to assess the effectiveness of a tumor-informed ctDNA assay in guiding treatment decisions for patients with stage II/III colorectal cancer (CRC) undergoing adjuvant chemotherapy treatment. Among the 295 patients evaluated, consisting of 130 with stage II and 165 with stage III CRC, 6.9% (n = 46) of stage II patients and 22.4% (n = 37) of stage III patients tested positive for minimal residual disease (MRD) through ctDNA analysis. The presence of MRD was significantly associated with worse disease-free survival (DFS) outcomes overall (HR, 20.8; 95% CI, 10.0–43.4, *p* < 0.0001), as well as within each subgroup (stage II: HR, 25.7; 95% CI, 6.8–96.7; stage III: HR, 18.1; 95% CI, 7.3–45.1). Patients who tested negative for MRD after surgery had a median DFS that was not reached, while those who tested positive had a median DFS of 15.98 months (range, 13.77–20.22). Among MRD-positive patients, those who received adjuvant chemotherapy had longer DFS compared to those in the observation group (18.7 vs. 6.7; HR, 3.9; 95% CI, 1.3–11.5; P = 0.01). Conversely, there was no observed benefit to DFS with adjuvant chemotherapy in the MRD-negative group (HR, 1.1; 95% CI, 0.3–3.9; P = 0.89).

Exosomes have indeed garnered significant attention in the field of precision medicine for their potential as biomarkers in various cancers, including CRC. Recent clinical trials and studies have explored their prognostic value and the molecular cargo they carry, such as proteins, lipids, and nucleic acids, which can provide critical insights into the tumor microenvironment and disease progression.

Exosomal microRNAs (miRNAs) have emerged as promising biomarkers for colorectal cancer (CRC), providing valuable insights into disease prognosis. Exosomes are small extracellular vesicles that carry various biomolecules, including miRNAs, which can influence cancer progression and patient outcomes. Several studies have demonstrated the prognostic significance of specific exosomal miRNAs in CRC.

There are several exosomal miRNA that have been studied in the context of CRC, and miR-21 is one of the most extensively studied exosomal miRNAs in CRC. Elevated levels of exosomal miR-21 have been associated with poor prognoses, including reduced overall survival and increased recurrence rates [227]. Other miRNAs are miR-1246, which high expression has been linked to advanced disease stages and poor survival outcomes in CRC patients. It has been proposed as a potential marker for monitoring disease progression [228]. Exosomal miR-200c has been identified as a prognostic marker, with higher levels correlating with better survival rates in CRC patients. This miRNA is involved in epithelial–mesenchymal transition (EMT) and metastasis suppression [229]. Exosomal miRNAs, such as miR-21, miR-1246, miR-200c, and miR-92a, hold significant prognostic value in colorectal cancer. They offer insights into disease progression, recurrence, and patient survival, making them valuable tools for enhancing the precision of CRC prognosis and potentially guiding therapeutic decisions. Continued research into these biomarkers is likely to further refine their clinical utility and integration into routine practice.

Circulating tumor cells (CTCs) have emerged as important prognostic biomarkers in colorectal cancer (CRC). Their detection and analysis provide valuable information about disease progression and patient outcomes. Notably, the prognostic significance of CTCs has been recognized in FDA-approved tests. The presence and number of CTCs in the bloodstream are associated with disease stage, treatment response, and overall survival. Higher counts of CTCs are generally linked to advanced disease and poorer prognosis. The CellSearch^®^ system is an FDA-approved test for detecting and enumerating CTCs in mCRC. It has been validated to provide prognostic information, where a higher number of CTCs is correlated with worse outcomes.

Multiple studies have shown that CTC count is an independent prognostic factor in CRC. For instance, Cohen et al. [230] demonstrated that patients with mCRC having more than three CTCs per 7.5 mL of blood had significantly shorter progression-free survival (PFS) and overall survival (OS) compared to those with fewer CTCs. Advances in technology have enabled not just the counting of CTCs but also their molecular characterization. Analyzing the genetic and phenotypic profiles of CTCs can provide insights into tumor biology, resistance mechanisms, and potential therapeutic targets [231].

CTCs can also serve as predictive biomarkers for monitoring treatment efficacy. A decrease in CTC count after initiating therapy is often indicative of a favorable response, while an increase may signal disease progression [232].

## 8. Ongoing Biomarker Guided Clinical Trials

As previously discussed, ctDNA-guided therapy involves utilizing ctDNA as both a predictive and prognostic biomarker in the treatment of mCRC. This innovative approach has been investigated in numerous clinical trials.

The first phase II trial to integrate ctDNA as a predictive biomarker in mCRC was the CHRONOS trial [233]. This study investigated the utility of blood-based identification of RAS/BRAF/EGFR mutations to tailor anti-EGFR rechallenge with panitumumab, focusing on objective response rate (ORR) as the main endpoint. The trial successfully met its primary endpoint, achieving a partial response rate of 30% and a stable disease rate of 63%. These ORR findings were comparable to other existing therapeutic options in the third line, indicating the potential significance of liquid biopsies in guiding treatment decisions. Additional trials investigating the utility of ctDNA in mCRC management include the REMARRY and PURSUIT trials [234]. These two-phase trials aim to assess the dynamics of plasma RAS status during subsequent treatments following refractoriness to anti-EGFR therapy in patients with mCRC harboring RAS/BRAF V600E wild-type tumors who have experienced progression following a previous response to anti-EGFR therapy. Furthermore, they aim to evaluate the efficacy and safety of rechallenge therapy with panitumumab plus irinotecan in patients without RAS mutations in ctDNA (plasma RAS negative).

As previously mentioned, the role of ctDNA has also been investigated in the adjuvant setting, as demonstrated by the BESPOKE trial [226]. This trial highlights the potential of ctDNA as a biomarker for selecting patients who would benefit the most from adjuvant therapy.

While these trials have produced conflicting results and primarily focused on anti-EGFR therapy rechallenge, they suggest that ctDNA could indeed be a valuable biomarker in the management of CRC.

A topic of interest regarding the optimal selection of first-line therapy in mCRC is the selection between anti-EGFR vs. anti-VEGF agents. This question has been raised by the CAIRO 5 trial [235], in which initial unresectable CRC with hepatic metastases have been treated based on tumor sidedness and RAS/BRAF mutational status. The trial reported that FOLFOXIRI and bevacizumab are preferrable in patients with right-sided or RAS/BRAF mutated tumors, and that in left-sided or RAS/BRAF wt tumors, the addition of panitumumab to FOLFOX or FOLFIRI did not show any benefit compared to bevacizumab. An ongoing phase III study [236] (LIBImAb; NCT04776655) aims to assess the superiority of bevacizumab compared to cetuximab with FOLFIRI in mCRC which exhibit RAS/BRAF wt status on tumor tissue, but appear mutated in plasma analysis. Patients with RAS/BRAF wild-type status undergoing targeted therapy will undergo the first liquid biopsy (LB), while those with RAS mutations will be randomly assigned in a 1:1 ratio to receive either FOLFIRI/CET (control arm) or FOLFIRI/BEV (experimental arm). Conversely, patients with RAS wild-type status at the first LB will receive treatment with FOLFIRI/CET for up to eight cycles. Patients who do not experience progression after eight cycles of treatment will undergo a second LB. If a RAS mutation is detected, patients will be randomly assigned in a 1:1 ratio to either continue FOLFIRI/CET or switch to FOLFIRI/BEV. If no mutation is detected, patients will continue FOLFIRI/CET outside the clinical trial.

The phase II trial PANIRINOX (NCT02980510) [237], which exclusively employs ctDNA for tumor molecular characterization, aims to assess the efficacy of FOLFIRINOX + Panitumumab versus mFOLFOX6 + Panitumumab in metastatic colorectal cancer (mCRC). The initial published findings support the notion that ctDNA serves as a viable alternative to molecular tissue analysis before initiating first-line anti-EGFR agents.

Currently, ctDNA is being researched as a potential biomarker for anti-EGFR rechallenge [238,239,240], progression anticipation [241,242], and selection of targeted therapy [243,244].

## 9. Conclusions

In this review, we highlight the importance of several types of biomarkers and the role they play in prognosis as well as treatment management of mCRC. From the conventional clinicopathological features to the cutting-edge molecular signatures, the prognostic and predictive determinants of CRC are multifaceted and dynamic.

While traditional factors such as tumor stage, grade, and lymph node involvement continue to hold prognostic significance and are routinely applied in day-to-day practice, emerging biomarkers, such as microsatellite instability (MSI), RAS/BRAF mutations, tumor-infiltrating lymphocytes (TILs) and HER2 mutations/overexpression, offer promising avenues for personalized management strategies.

In summary of the above-presented data, we believe the following: RAS/BRAF mutation testing should be conducted in all cases of metastatic colorectal cancer (mCRC). While there is evidence supporting its prognostic value in stage III CRC, we do not deem RAS/BRAF testing to be cost-effective in stage I–III CRC. MSI status should be determined regardless of the stage; both immunohistochemistry and RT-PCR (reverse transcription polymerase chain reaction) are valid methods for testing with almost no difference between them. The significance of MSI status lies in its crucial role in managing mCRC, as current data favors ICI (immune checkpoint inhibitors) treatment as the preferred first-line approach. Recently POLE/POLD1 mutations have been incorporated into the NCCN guidelines, based on findings that indicate the effectiveness of ICI treatment in mCRC with these mutations.

While tumor microenvironment analysis is intriguing, supported by numerous retrospective studies on various plasma biomarkers, Immunoscore stands out as the sole well-validated pathological score to guide adjuvant treatment selection in stage II–III CRC patients.

HER-2 mutation/amplification testing emerges as a novel and compelling biomarker. It not only predicts the efficacy of HER2-targeted therapy but also influences the effectiveness of anti-EGFR targeted treatment. Studies indicate that mCRC patients with RAS/BRAF mutations do not benefit from anti-HER2 treatment with trastuzumab, pertuzumab, or lapatinib. Conversely, anti-EGFR treatment is ineffective in HER2-mutated/amplified cases. Moreover, the advent of liquid biopsy, particularly circulating tumor DNA (ctDNA), presents a paradigm shift in the non-invasive assessment of disease burden and treatment response, offering real-time monitoring and dynamic insights into tumor evolution. An intriguing exception to this principle arises from the role of trastuzumab deruxtecan in HER2-overexpressing/amplified patients, as illustrated in the DESTINY-CRC02 trial. Despite the response rate being lower compared to RAS/BRAF wild-type patients, it remains significant (28.6% vs. 39.7%). This can be attributed to the mechanism of the antibody–drug conjugate (ADC), which utilizes HER2 as a receptor to internalize the cytotoxic agent. Considering this, HER2 testing might be considered regardless of RAS/BRAF status in mCRC patients who are refractory to other lines of therapy.

Amidst promising advancements, however, several challenges persist, including the need for standardization and validation of biomarker assays, integration of multi-omics data, and optimization of therapeutic strategies tailored to individual tumor biology.

Moving forward, collaborative efforts across disciplines, robust clinical trials, and advancements in technology will be pivotal in harnessing the full potential of prognostic and predictive determinants, ultimately enhancing patient outcomes and shaping the future landscape of CRC management.

## Data Availability

The data presented in this study are available on reasonable request from the corresponding authors.
